# Expanding Social, Psychological, and Physical Indicators of Urbanites’ Life Satisfaction toward Residential Community: A Structural Equation Modeling Analysis

**DOI:** 10.3390/ijerph18010004

**Published:** 2020-12-22

**Authors:** Chuanyu Peng, Guoping Yuan, Yanhui Mao, Xin Wang, Jianhong Ma, Marino Bonaiuto

**Affiliations:** 1 School of Public Affairs and Law, Southwest Jiaotong University, Chengdu 610031, China; Chuanyu.Peng@swjtu.edu.cn (C.P.); guoping.yuan@my.swjtu.edu.cn (G.Y.); wx1996@my.swjtu.edu.cn (X.W.); 2 Psychological Research and Counseling Center, Southwest Jiaotong University, Chengdu 610031, China; 3 Department of Psychology and Behavioral Sciences, Zhejiang University, Hangzhou 310028, China; jhma@zju.edu.cn; 4 CIRPA—Centro Interuniversitario di Ricerca in Psicologia Ambientale, Dipartimento di Psicologia dei Processi di Sviluppo e Socializzazione, Sapienza Universitá di Roma, 00185 Roma, Italy; marino.bonaiuto@uniroma1.it

**Keywords:** flow, PREQIs, green area, community engagement, social capital, community identity, life satisfaction

## Abstract

Attention on, and interest in, life satisfaction has increased worldwide. However, research on life satisfaction focused toward the urban dwellers’ residential community is mainly from western countries, and the limited research from China is solely focused on the geriatric population via a narrowly constrained research perspective. This study, therefore, aimed to investigate urbanites’ life satisfaction toward their community, combining the psychological (behavioral community engagement, mental state of flow, and cognitive community identity), physical (PREQIs-perceived residential environment quality indicators: e.g., green area), and social perspectives (social capital). The proposed conceptual model was tested on a regionally representative sample of 508 urban community residents in the city of Chengdu, Sichuan province, China. Data were analyzed via a structure equation modelling approach in AMOS software. Findings suggested that all of the psychological, physical and social factors contributed to a prediction of life satisfaction. Specifically, social capital mediated the path from community engagement and flow to life satisfaction, and community identity mediated the path from flow experience and green area to life satisfaction. Additionally, social capital contributed to predict life satisfaction through its influence on community identity. Findings provide suggestions for urban designers and policymakers to focus on creating an urban community equipped with green area, which helps to promote physical activities that are flow-productive, to enhance residents’ identification to their residential community and, therefore, increase life satisfaction.

## 1. Introduction

Life satisfaction, as a subjective evaluation of overall quality of life [1,2], is one of the core elements of well-being and has been of great interest to psychologists [3,4], it is also a central construct within positive psychology [5]. Life satisfaction refers to a judgmental process in which individuals assess the quality of their lives on the basis of their own unique set of standards [6], and attention on, and interest in, life satisfaction has increased worldwide. Studies have shown that people with high life satisfaction experience many positive outcomes in their lives, which are usually related to income, education, health, employment status, social capital, self-esteem, and so forth [7,8]. However, research on life satisfaction towards the dwellers’ residential community of place is mainly from western countries [9,10,11], and the sparse literature from China is solely focused on a specific age group of the elderly population [10,12]. Therefore, research on life satisfaction of urbanites of all populations geared toward residential communities is emerging and urgently needed from the perspective of both positive psychology and environmental psychology. This is particularly true nowadays, especially in a residential confinement contingency such as during the COVID-19 pandemic when residency settings (home, building, neighborhood, city, province, and the country) became pervasive given a nonmobile human life. Our primary aim was to investigate the possible factors that may contribute to the understanding of the urbanites’ life satisfaction from the psychological, social, and physical perspectives.

### 1.1. Green Area that Shaped Our Residential Community

There has been considerable effort from environmental psychologists [13,14,15,16] in the study of the residential environmental quality that is associated with a human being’s psychological wellness and life satisfaction. Accordingly, they have invented specific scales such as the perceived residential environmental quality indicators (PREQIs) that have been tested and validated in a variety of language and cultural contexts [12,17,18]. The aim of the PREQIs is to grasp specific cognitive evaluations with respect to a range of certain residential features from the residents’ points of view [19]. For instance, when evaluating the spatial feature of the residents’ community on green areas, they use items like “*there are enough green areas in this residential neighborhood*”. The great benefits of green areas to well-being and life satisfaction have been thoroughly scrutinized among environmental psychologists [20,21,22,23].

Green area, or green space, plays an important role in residents’ lives in times of urban expansion [24,25]. Green area is an important recreational facility of residential areas and includes street trees, squares with green elements, parks, gardens, and forest plantations within residential communities [18,25,26]. Previous research has shown that there is a positive correlation between exposure to residential green area and the physical and mental health of residents. Green area may provide an inviting setting for walking, jogging, and cycling activities, thereby promoting physical health. It can also reduce psychological stress, anxiety, and depression to promote mental health [27,28,29]. Additionally, green area provides a platform for social activities, creating opportunities for outdoor social interaction among residents (e.g., engaging in leisure and sport activities, meeting old friends, or making new acquaintances) [30,31]. Evidence also shows that a child’s connection to nature influences their intention to participate in nature-based activities in the future, that is to say, the closer the connection to green area, the more it promotes children to form environmentally friendly behaviors [32]. Further, environmental psychologists found that spending 120 min a week in residential green area is associated with good health and well-being [21]. Thus, green area within a residential community—a place people live in everyday—can help people live in a healthier and more natural way in the city [23].

Identifying a residential community as a specific geographically located place, where people live their everyday life, enables the provision of public spaces for residents’ individual activities and social interactions. It seems that the quality of the residential community—to some extent—determines life satisfaction, especially for those who are over 50 years of age [33] and for those who are retired [12]. Extensive studies have also found that the quality of the residential community is of key importance for a dweller’s subjective well-being [34]. However, in China, a country of fast urbanization with huge population and a densely inhabited community, research on green areas embedded in communities for promoting life satisfaction within the Chinese urban context is sparse, highlighting the need for in-depth exploration. Residential community-located activities in which residents are regularly engaged help increase perceptions of a positive, intrinsic, and enjoyable experience of flow [35].

### 1.2. Flow: Life of Engagement in Our Residential Community

Flow, as a foundational theory in positive psychology, has been receiving great attention since it was first introduced [36]. It is a mental state related to happiness and well-being, encompassing the feeling that there is something to live for. Flow can be characterized by high involvement, deep concentration, intrinsic motivation, and the perception of exhilarating challenges matched by adequate personal skills [37,38], and it also describes a highly positive state of the individual, who experiences intense engagement and enjoyment from various types of self-defining activities [35,39]. By reviewing prior studies, flow experience is summarized by the following characteristics: (1) devoting oneself to the things they are doing, (2) the combination of action and consciousness, (3) the loss of self-consciousness, (4) the balance between skills and challenges, (5) clear and realizable goals, (6) immediate and clear feedback, (7) a sense of control over one’s behavior, (8) distorted time perception, and (9) experience of the activity is intrinsically rewarding [40,41,42]. As a universal activity experience emphasizing the purpose and meaning of life [43,44], flow has been applied in a variety of fields and research contexts such as workplace, education, marketing, and sports/leisure [45,46,47]. However, the application of flow in community research can barely be traced in research records.

### 1.3. Community Engagement and Community Identity

Community engagement can be considered as an active involvement in activities that are intrinsically social. It is a dynamic relational process that facilitates communication, interaction, involvement, and exchange. A resident’s community engagement includes participating in community activities, investing in community operations, interacting with community members, voting, and volunteering [10,48]. The main purpose of participation is to benefit the community members: more participation within such an institutional arrangement (i.e., community) decreases individual anxieties, apprehensions, and limitations on the one hand, and increases social mobility towards social empowerment on the other hand [49]. Furthermore, community members would have a deeper and more specific understanding of community organizational behavior through engaging in the above-mentioned community activities, and build up close connections with each other [48]. In addition, community-level engagement may help improve the level of public welfare, promote public trust, eliminate health disparities, alleviate poverty, protect the environment, and so on [50]. Community engagement can be closely related to life satisfaction of its residents, for instance, the more frequent the participation within community-based activities, the more satisfaction there is among community-dwelling older adults [51].

Identity is “*a set of meanings attached to the self that serves as a standard or reference that guides behavior in situations*” [52]. Community identity reflects the degree of a resident’s recognition of the neighborhood community in which she/he lives [53], and “*place identity*” refers to a specific place contribution in the definition and development of her/his own identity, in both physical and social terms (such as in the case of a urban residential community) [54]. The concept of place identity can be dated back to Proshansky’s work in the 1970s and 1980s, from which “*place identity*” was thought to convey the sense of personal attachment to geographically locative places, or specific spatial locations, as it is “*a person acquiring a sense of belonging and purpose which gives meaning to his or her life*” [55,56]. The tripartite model of place attachment provides a theoretical framework for understanding a three-dimension relationship among person, place, and psychological process [57]: the person dimension deals with who is attached, and whether the attachment is based on an individual or the collective; the place dimension comprises varying aspects of place, including spatial or physical, and social features; and the psychological process dimension describes the affective, cognitive, and behavioral manifestations of the attachment. One’s positive engagement with the community place may affect their affective, cognitive, and behavioral bonds to such place [58], whereas the absence of certain urban landmarks (e.g., green area) of that place may result in changes in one’s attachment [59,60], and such changes may lead to changes of community identity. From the perspective of social identity theory [61], when a community resident regards himself or herself as one of the community members, he or she may adopt a positive stereotype defined by community [62], and this social self-stereotyping process may therefore lead to community identity. Moreover, residential experience not only increases the opportunities for contact among residents, but also provides a temporal background for connecting important life events within their communities [63]. Taken together, a community can be conceived, and it functions as both a geographically located place and a social environmental reality that facilitates interactions, through which the connection between self and environment is established and maintained [35], thereby community identity is established and maintained.

### 1.4. Social Capital as a Resource of Residential Community

Social capital can be traced back to Bourdieu’s definition: “the aggregate of the actual or potential resources which are linked to possession of a durable network of more or less institutionalized relationships of mutual acquaintance or recognition” [64]. This definition focused on social networks. Later, social capital was defined by its function, referring to a variety of different entities instead of a single entity comprising two elements in common: “such two elements consist of some aspect of social structures, and they facilitate certain action of actors—whether persons or corporate actors—within the structure” [65]. Baker defined social capital as “a resource that the individuals derive from specific social structures and then use to pursue their interests; it is created by changes in the relationship among the individuals” [66]. Putnam referred to social capital as “features of social organizations, such as networks, norms, and trust, that facilitate action and cooperation for mutual benefit” [67]. Taken together, social networks, social norms, and trust are common elements for social capital [68], thus, social capital can be grouped into three broad dimensions: social networks; social norms, and social trust [69].

In our work, the study of social capital with respect to the context of the community is defined as a kind of social resource existing in the social network relationship embedded within the community [70,71]. This kind of resource is regarded as one that can adjust people’s behavior to achieve a specific goal and generate return on investment, including community communication, community trust, community participation, community identity, and so forth. Some evidence suggests that community identity and life satisfaction of the Chinese migrant workers are associated when the provided services are adequate [72]; in some sense, the provided service can be regarded as community social capital, which may facilitate life satisfaction. Moreover, the well-equipped and high standards of residential communities or neighborhoods investigated by Bonaiuto and colleagues also provided resources for community social capital [13,18]. Prior evidence has demonstrated that the perceived residential environmental quality indicators are correlated with neighborhood attachment [19]. It can be easily inferred that physical resources equipped within a residential community can also function as a kind of social capital, which may help a community member’s attachment to their residential place.

## 2. Hypotheses Development

### 2.1. The Relationship between Community Engagement, Flow Experience, Green Area, and Life Satisfaction

The relationship of life satisfaction with community engagement can be traced in numerous records. For instance, engaging in leisure-time physical and social activities was found to be positively related to life satisfaction among 2344 community-dwelling older adults in Brazil [51]. Social engagement was positively associated with life satisfaction of 4245 older residents in China [10]. Actively engaging in more than two social group activities was associated with higher life satisfaction from 136 persons with musculoskeletal impairments who belonged to disabled people’s groups [73]. Studies investigating the key determinants of life satisfaction from 466 older individuals living in the community in Dublin Ireland suggested that social and physical engagement could be targeted in a bid to improve life satisfaction for an elderly community-dwelling population [11]. On these basic points and referring to the urban residential community in China, we propose the following hypotheses:

**Hypothesis 1 (H1a)**: *Community engagement is positively associated with life satisfaction (confirmative hypothesis).*

The relationship between life satisfaction and flow experience can also be found in limited work. For example, flow at work was found to be predictive for life satisfaction and work longevity among 133 Filipino guidance counselors [74]. Flow significantly predicted the life satisfaction of 480 primary school teachers from different regions of Croatia [75]. All of the nine dimensions of flow had a positive correlation with life satisfaction among 452 Slovenian participants, who were either elite musicians or top athletes [76]. Study from 119 community-living elderly Japanese living in rural areas demonstrated that those who participated in “*meeting for the elderly*” group activities at a nursing home in Kagawa Prefecture, Japan, when having experienced more flow, reported a better quality of life in physical health, which may bring them life satisfaction [77]. Findings from 517 participants from South Korea via structural equation modeling approach indicated that flow from restaurants’ social networking sites (SNS) positively influenced SNS satisfaction, therefore increasing offline purchase intentions [78]. Data from a group of young Norwegian adults analyzed by means of structural equation modeling demonstrated that flow was associated with the satisfaction of life [79]. Though the application of flow to study urban community is sparse, the above studies in some sense provided an empirical basis for our hypothesis:

**Hypothesis 1 (H1b)**: *Flow is positively associated with life satisfaction (confirmative hypothesis).*

**Figure 1:**
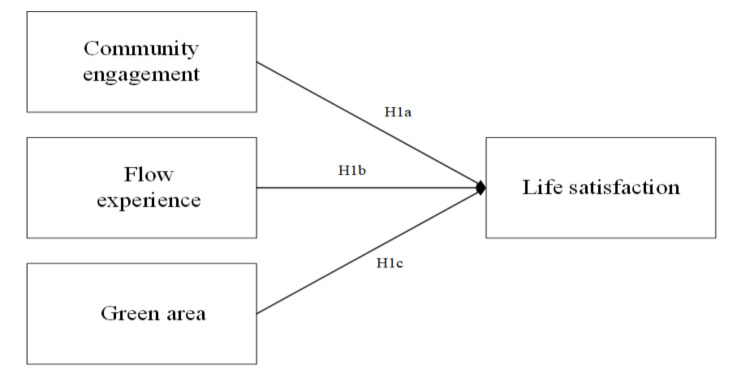
The basic model.

The physical environment created by green area can reduce the exposure to air pollution, noise, and heat, which closely affects the quality of life of the inhabitants [80,81] Additionally, many studies provide evidence for the importance of living in urban green areas for reducing mental stress and increasing well-being [20,82]. As urbanites confront heightened pressure from work and family or other domains, a need for recovery is mandated [25,81]. Green area is not only recognized as a suitable setting for running and jogging, but also for its potential in the alleviation of self-reported nervous problems and medication use [83]. In this sense, green area is considered as an important means by which to improve the quality of life of urbanites [84]. Indeed, empirical evidence has found that green area directly affects people’s physical health and psychological well-being in a positive way, which may improve life satisfaction of residents [85]. Based on the above reasoning and its application to urban residential community, we propose the following hypothesis (see Figure 1):

**Hypothesis 1 (H1c)**: *Green area has a positive influence on life satisfaction (confirmative hypothesis).*

### 2.2. The Relationship between Community Engagement, Flow Experience, Green Area, and Life Satisfaction: Social Capital as a Mediator

**Figure 2:**
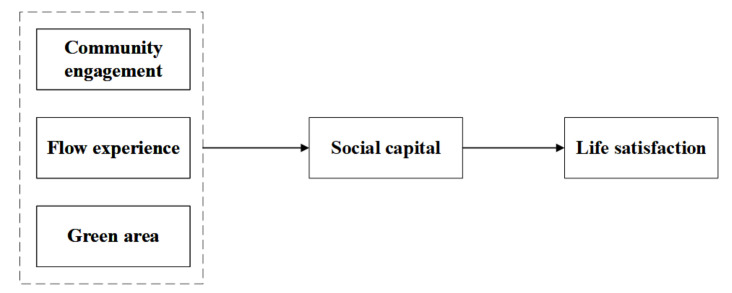
The mediation model: social capital as a mediator.

Social capital at the community level emphasizes the ability of the community to provide its members with opportunities to increase personal and family resources. The core theme of social capital is interpersonal relationships and reciprocal value [71]. Extensive research has found the association of social capital with constructs like community engagement [48,86], flow experience [87], green area [88,89], and life satisfaction. Empirical evidence showed that the neighborhood-based social capital and residents’ life satisfaction are positively associated [90]. In addition, social capital of the *Wechat* network was found to be a significant predictor of life satisfaction of Chinese overseas students in Germany [46], and social capital was predictive for life satisfaction of a special group of elderly migrants [88]. On these bases and in reference to our residential community context, we propose the following group of hypotheses (see Figure 2):

**Hypothesis 2 (H2a)**: *Social capital mediates the relationship between community engagement and life satisfaction (innovative hypothesis).*

**Hypothesis 2 (H2b)**: *Social capital mediates the relationship between flow experience and life satisfaction (innovative hypothesis).*

**Hypothesis 2 (H2c)**: *Social capital mediates the relationship between green area and life satisfaction (innovative hypothesis).*

### 2.3. The Relationship between Community Engagement, Flow Experience, Green Area, and Life Satisfaction: Community Identity as a Mediator

The direct relation of community identity with respect to community engagement can only be found in limited records. However, some indirect evidence can be traced, for instance, an investigation of 338 players who have experience in engagement in online communities through virtual experience suggested that consumer engagement was associated with social identity, which, in some sense, related closely to the online community [91]. School engagement positively predicted an information-oriented identity [92], and civic engagement related to personal identity and social identity in late adolescents and emerging adults [93]. These studies suggested that community engagement may impose an impact on community identity. The direct relation of community identity with respect to life satisfaction can hardly be traced. However, some implicit findings may provide evidence. For example, findings from 1087 rural-to-urban migrant workers in Shenzhen, China indicated that community service use is positively correlated with both identity integration and migrant workers’ life satisfaction. Moreover, identity integration served as a partial mediator between community service use and life satisfaction [72]. This indirectly suggested a relationship between community identity integration and life satisfaction, and provided help for the following assertion:

**Hypothesis 3 (H3a)**: *Community identity mediates the path from community engagement to life satisfaction (innovative hypothesis).*

The direct relations between community identity and flow are also hard to trace. However, some prior implicit evidence can be found: for instance, there were associations between social identity and flow when engaging in specific social group activities [94], there were links between place identity and flow when engaging in place-located activities [35], and there were associations between personal identity and flow when engaging in individualized self-defining activities as well [94]. An analysis of the personal narrative of flow experiences across three elite sportsmen within their particular sport in autobiographical contexts showed a close relationship between flow and athletic identity [95]. The above-mentioned work actually provided the primary quantitative evidence for the link between flow and identity at different levels. As community functions as both a specific social group and a geographically located place, community identity with respect to flow experience (though, to our knowledge, not yet studied) and their underlying potential link may exist. Together with prior inference on community identity and life satisfaction, we therefore propose the following hypothesis:

**Hypothesis 3 (H3b)**: *Community identity mediates the path from flow to life satisfaction (innovative hypothesis).*

**Figure 3:**
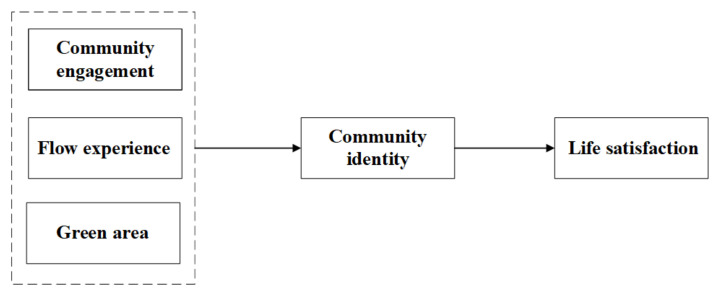
The mediation model: community identity as a mediator.

The implicit association between green area and community identity can be traced in existing literature: for instance, a study of 328 inner-city dwellers in two European cities in Ljubljana and Edinburgh reported positive and significant effects of involvement in, and appreciation of, green areas on sense of place, in which place identity is involved [31]. Findings from a community in Maryland, U.S.A, demonstrated that community green areas with natural features and open spaces play a particularly important role in community identity, as they foster pedestrianism and increase the likelihood of social interactions [96]. There are also few studies about the effect of community identity on life satisfaction of residents [72]. Together, with the prior section, providing firm evidence on the effect of green area on life satisfaction, we therefore put forward the following hypothesis (see Figure 3):

**Hypothesis 3 (H3c)**: *Community identity mediates the path from green area to life satisfaction (innovative hypothesis).*

### 2.4. The Relationship between Social Capital and Community Identity

Social capital as a kind of resource and power was found to be positively associated with identity [71,97]. For instance, findings from a group of Iranian graduate students suggested that the online social capital and social networking created an environment for their professional identity construction [98]. A qualitative study where 26 Danish and 11 Australian university students were interviewed suggested that creating opportunities for social interaction with educators at universities could help facilitate the generation of bridging social capital, which, in turn, is essential for students’ professional identity development [97]. A study of 344 Taiwan college students demonstrated that the fair and reciprocal exchange of social capital among members could facilitate maintaining or enhancing the development of social network relations, as well as the formation of group identity [99]. Findings from 2092 seventh-grade Korean students via SEM showed that both family social capital and school social capital are important to predict a youth’s career identity irrespective of their socioeconomic status. Family social capital had direct and indirect effects on a youth’s career identity, while school social capital had direct effects on a youth’s career identity [100]. A cross-sectional study of 374 low-income, rural, African American adolescents suggested that adolescents who have stronger commitments to their interpersonal identities will report better social capital quality; social capital is particularly important for the well-being and future opportunities of African American adolescents living in low-income families [101]. The above studies provided support for the inclusion of social capital in the extended residential community context, since the resources obtained from residential community as a social capital may also be beneficial for the function of their community identity, therefore we propose the following:

**Hypothesis 4 (H4)**: *Social capital has a positive influence on community identity (confirmative hypothesis).*

**Figure 4:**
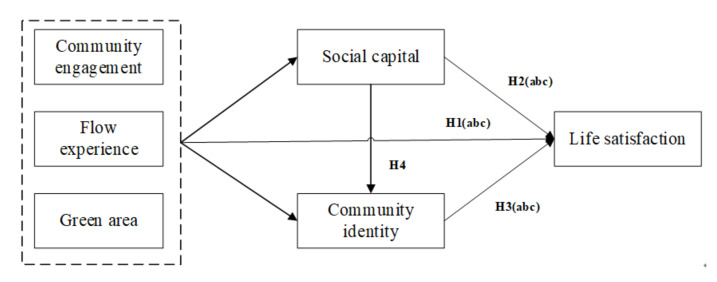
The conceptual model.

The general conceptual model of this study is therefore as follows (see Figure 4):

## 3. Methods

### 3.1. Participants and Context

**Figure 5:**
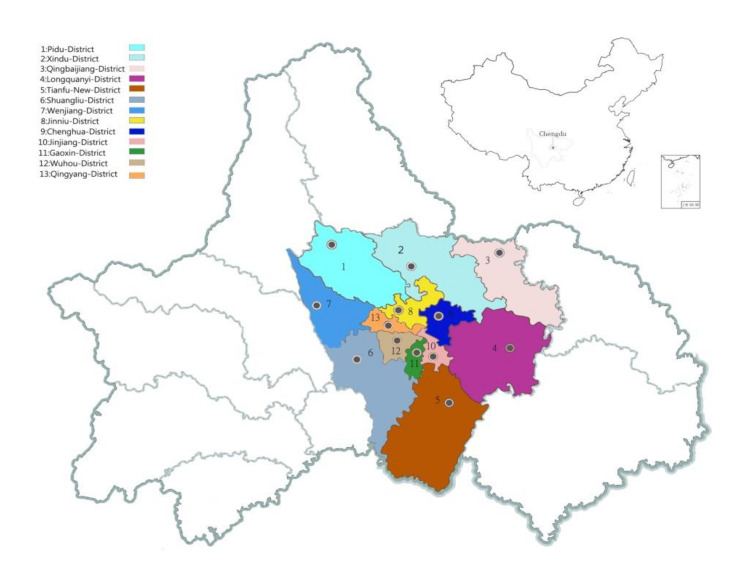
Sampling administrative district of neighborhood communities in Chengdu.

A total of 1000 residents were contacted, of which 520 responded (response rate was 52%). Excluding those individuals who were either inattentive or for whom there were missing data on crucial study variables, a final sample of 508 participants remained (completion rate was 50.8%). Their social demographic features are presented in Table 1. Participants were living in urban communities within 13 districts in Chengdu (see Figure 5), which are characterized by their different distances to the city center. Chengdu is located in central Sichuan Province in southwest China; it is known as the “*Country of Heaven*” and is associated with a Chinese national symbol of the Giant Panda. Chengdu is also one of the most important economic, financial, commercial, cultural, transportation, and communication centers in Western China. Above all, it is considered as one of the most livable cities (top 5) in China [102], especially with a recent “*park city*” campaign for “*greened*” with flowers and plants around the city [103].

### 3.2. Measures

In the present work, answers regarding each measure were registered on a 5-point Likert-type scale ranging from 1 “*strongly disagree*” to 5 “*strongly agree*”.

*Satisfaction with life*. To measure participants’ overall perception of life satisfaction toward the residential community where they regularly live, a short form (5 items) of the satisfaction with life scale developed by Diener and colleagues [104,105] was provided. Items include *“In most ways my life is close to my ideal”*, *“The conditions of my life are excellent”*, *“I am satisfied with my life”*, *“So far I have gotten the important things I want in my life”,* and *“If I could live my life over, I would change almost nothing”*. Such a scale has been utilized to study subjective happiness in a variety of previous works [106,107]. Cronbach’s α regarding the present sample was 0.857 (Cronbach’s α > 0.7 is acceptable, and <0.35 is considered to be low reliability and must be rejected) [108].

*Flow*. To remain consistent with our prior investigation on flow [94,109,110], we followed prior research on flow by Waterman and colleagues [111,112,113], adapted into a 9-item flow state scale based on flow theory [36] to measure residents’ perceived flow within their community. Items were phrased as completions of a common stem anchored by 1 “*strongly disagree”* to 5 “*strongly agree”,* with 3 “*moderately agree”* in the middle: “When I regularly engage in this activity ____ (e.g., jogging, morning exercise, dance) within my residential community, ____”, items to be completed were as follows: (1) *“I feel I am competent enough to meet the high demands of the this activity”*, (2) *“I do this activity spontaneously and automatically without having to think”*, (3) *“I have a strong sense of what I want to do”*, (4) *“I am clear about how well I am doing”*, (5) *“I am completely focused on the present activity at hand”,* (6) *“I have a feeling of total control”*, (7) *“I am not worried about what others may think of me”*, (8)*“The way time passes seem to be so quick”*, and (9) *“The activity is extremely rewarding”*. Cronbach’s α for the present sample was 0.757.

*Community engagement*. We utilized a 5-item community engagement scale from prior works [114,115]; this scale was developed to measure community members’ civic involvement and participation in community activities. These five items are *“I collaborate in organizations and associations in my residential community”*, *“I take part in social activities in my residential community”*, *“I take part in some social or civic groups in my residential community”*, *“I respond to calls for support in my community”*, and *“I don’t take part in socio recreational activities in my residential community”*. Cronbach’s α yielded from the present dataset was 0.904.

*Green area.* To assess residents perceived green area within their community, we applied a 6-item scale, one negative and five positives, developed by Bonaiuto and colleagues [13,14]. Items are (1) *“There are enough green areas in this community”*, (2) *“There are green areas for relaxing in this neighborhood”*, (3) *“In this neighborhood green areas are in good condition”*, (4) *“The green areas are too small in this neighborhood”*, (5) *“There is at least a garden/park where people can meet in this neighborhood*”, and (6) *“The green areas are well-equipped in this neighborhood (lighting, driveways, benches, waste bins, etc.)”*. This scale was widely used in the literature for examining residential environmental quality, both in China and abroad, and indicated good reliability and validity [12,18,116]. Cronbach’s alpha regarding the present sample was 0.889.

*Social capital*. We adapted a 5-item scale to measure structural social capital of residents within community, revised by De Silva and colleagues [117] according to Adapted Social Capital Tool (A-SCAT) [118]. All social capital measures in De Silva’s study refer to the “community” as local administrative boundaries [117], which is consistent with our selected administrative areas within this study. These items are (1) “*In the last 12 months, I have been an active member of the groups in my community*”, (2) “*In the last 12 months, I have received emotional help, economic help, or assistance in helping I know or do things from the group*”, (3) “*In the last 12 months, I have received help or support which can be emotional help, economic help, or assistance in helping I know or do things*”, (4) “*In the last 12 months, I have joined together with other community members to address a problem or common issue*”, and (5) “*In the last 12 months, I have talked with a local authority or governmental organization about problems in my community*”. This scale was widely used throughout the literature [119]. Cronbach’s alpha regarding the present sample was 0.9, which indicated good reliability.

*Community identity*. Considering that the residential community was considered as a physical area or place, the measurement of the residents’ place identity—a subcomponent of self-identity referring to the person and place connection between a physical setting and personal identity [56,120]—can be applied to a specific place like a residential community [13,121,122]. Therefore, we adapted a 6-item place identity scale used by prior work [123] into the context of residential community. Items were phrased as (1) *“I feel my residential community is a part of me”*, (2) *“My residential community is very special to me”,* (3) *“I identify strongly with my residential community”*, (4) *“I am very attached to my residential community”*, (5) *“Visiting my residential community says a lot to me”*, and (6) *“my residential community means a lot to me”.* Cronbach’s alpha for testing community identity in the present sample was 0.867.

### 3.3. Procedure

Before the formal questionnaire, we invited 10 urbanities from different neighborhood communities located in different administrative districts in Chengdu to take part in face-to-face interviews, asking them questions like “*which factors may affect life satisfaction toward your neighborhood community?*”, and “*did you enjoy engaging in any community activities?*”. Among all factors that the participants reported, the top 5 were chosen as our study variables, of which “green areas” was selected for its validation in a different language and cultural context in China. Subsequently, all questionnaire items regarding each variable were translated from original English into Chinese, then back-translated into English to ensure the accuracy of the translation and enable respondents to understand the items correctly. The online version of the questionnaire was created by putting all items on an EFS software survey platform. The survey link was sent to the potential respondents via *Wechat* and *QQ* (popular instant messaging systems that are similar to Skype and are widely used in China) by our researchers in order to obtain their participation. Later, a pilot survey with 269 participants was carried out before the formal data collection. Then, according to the results of this pilot, we adjusted the items of the questionnaire, removed semantic ambiguity and duplicate items, and formed a final formal online questionnaire. Finally, participants were asked to accept the invitation to complete the questionnaire with reference to their own residential community (see Figure 5). Ethical approval was not required since the survey did not bring any psychological or physical harm to participants. The data gathering phase ran from 30 March to 1 June, 2020.

### 3.4. Data Analytic Strategy

Data analyses were conducted in IBM SPSS (version 26.0) and AMOS (version 24.0). **First**, the Kaiser–Meyer–Olkin (KMO) coefficient and the Bartlett test were conducted in SPSS to determine whether the questionnaire is reliable and the data obtained were consistent with the factor analysis. The value of KMO above 0.5 is acceptable, and above 0.7 indicates it is suitable for factor analysis [124]. **Second**, confirmatory factor analysis (CFA) for the constructs of interest, conducted via AMOS, was applied to validate the factorial structure of questionnaire scales. A set of fitting indices of CFA models (and relative cut-off values for acceptable fit) was adopted [125], of which, the root-mean-square error of approximation (RMSEA) indicated how well the model would fit the population covariance matrix (RMSEA < 0.05 indicating close approximate fit, 0.05 < RMSEA < 0.08 suggesting reasonable error of approximation, and RMSEA above 0.10 indicating it should be rejected) [126]. The standardized root-mean-square residual (SRMR) was used to measure the overall difference between the observed and the predicted correlations, with a cut-off value below 0.10 generally considered favorable [127]. The comparative fit index (CFI) was used to assess the relative improvement in fit of the researcher’s model compared with a baseline model, with a cut-off value of 0.95 [128]. Other indices included goodness-of-fit index (GFI), incremental fit index (IFI), and non-normed fit index (NNFI), with values of these indices above 0.9 indicating good fit [129,130]. Finally, the chi-square/degrees of freedom ratio was used, with a ratio between 1 and 3 indicating good fit (Hsu et al., 2012). **Subsequently**, a structure equation modeling (SEM) approach was utilized to test our proposed conceptual model within AMOS.

## 4. Results

### 4.1. Validity and Reliability of the Measurement Model

In order to ensure the reliability and validity of the measurement model, we conducted a Kaiser–Mayer–Orkin (KMO) test and a Bartley spherical test. According to suggestions, a KMO > 0.5 with significance level of *p* < 0.05 would indicate that the questionnaire is of good structural validity [124]. As shown in Table 2, the KMO values for community engagement (CE), green area (GA), community identity (CI), social capital (SC), and life satisfaction (LS) were all above 0.8 (*p* < 0.001), and the value for flow was above 0.7 (*p* < 0.001), indicating a good fit of our measurement model. Additionally, the factor loadings were all greater than 0.5, indicating that the extracted common factors were highly representative of each variable, and the overall performance was good [131]. The average variance extraction (AVE) values of community engagement, green area, community identity, social capital, and life satisfaction were all greater than 0.5, and the AVE value of flow was extremely close to 0.5, reaching a convergence effect [131]. It is generally believed that the correlation coefficient between variables is highly correlated in the range of 0.5 and 1. The value of composite reliability (CR) for each measurement variable was greater than 0.6 [132]. Therefore, the measurement constructs used in the study are of good reliability.

### 4.2. Descriptive Statistics and Correlations

Descriptive statistics regarding all study variables with means, standard deviations, and correlations are presented in Table 3. As indicated, participants’ mean scores on community engagement, green area, community identity, and life satisfaction are respectively higher than the midpoint of 2.5, while the mean score of social capital is slightly lower than 2.5, indicating that most of the respondents in this study hold a positive attitude towards life satisfaction.

The results of Pearson bivariate correlation coefficients among six variables are presented in Table 4. As displayed, they were all positively and significantly correlated, except for a single case on the relations between community engagement and green area. It is worth noting the positive and significant correlations of life satisfaction with other study variables, indicating that engaging in activities within the community equipped with green areas is associated with flow, community identity, and life satisfaction.

### 4.3. Test of Hypotheses via SEM

To evaluate the goodness-of-fit of the default model, a series of fit indices were used, as shown in Table 5. The index of X^2^/*df* of the basic model was 1.975, which in the mediation model was 1.855 (1 <  X^2^/*df* < 3, *p* < 0.001). Values of the SRMR of the basic model and the mediation model were all less than 0.1. RMSEA values were 0.044 and 0.041, respectively, both below 0.08. The values of Goodness-of-Fit Index (GFI), Incremental Fit Index (IFI), Non-Normed Fit Index (NNFI), and Comparative Fit Index (CFI) in these two models were all above 0.9. Additionally, the values of Akaike Information Criterion (AIC) and Expected Cross-Validation (ECVI) of the default model were lower than the values of the saturated model (the value of AIC and ECVI in the basic model were 342.000 and 0.675, respectively, and in the mediation model they were 812.000 and 1.511) and the independence model (the values of AIC and ECVI in the basic model were 5149.642 and 10.157, respectively, and in the mediation model they were 9186.656 and 18.120) [129]. Therefore, these indices indicated that the default model fitted well.

Table 6 reports the regression weights of our basic model, and the results show that flow, community engagement, and green area had a positive and significant effect on life satisfaction, which supports Hypotheses H1a, H1b, and H1c.

Table 7 and Table 8 report the regression coefficients and the test of bootstrap results via the structure equation model in AMOS, considering social capital and community identity as mediators. As indicated in Table 7, the associations for community engagement, flow, green area, and life satisfaction were no longer statistically significant when community identity and social capital were considered as mediation variables. Together with Table 8, it can be seen that social capital and community identity mediated the path to life satisfaction.

**Figure 6:**
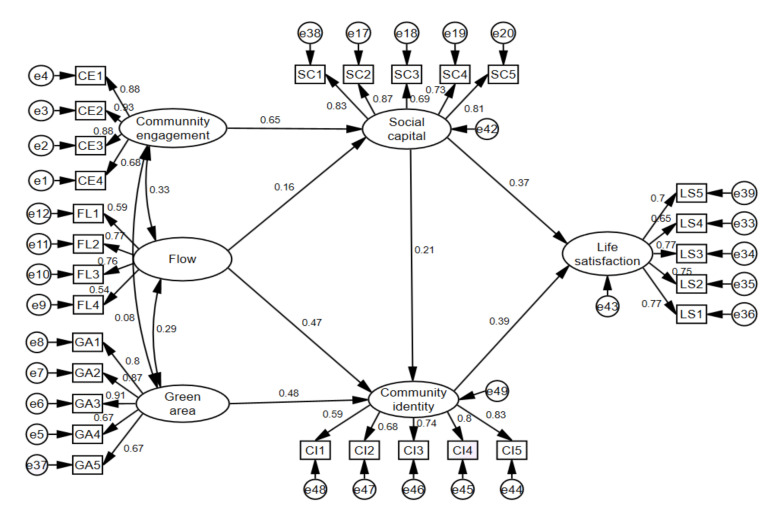
Results of the validated model.

We used the bootstrap method via AMOS to analyze the significance of the mediation effects of social capital and community identity, with a sample number of 5000 and a confidence interval of 95%. As indicated, flow and residents’ community engagement exhibited direct influences on social capital. Flow, also, together with the green areas, directly influenced residents’ community identity. Social capital had a direct and significant impact on community identity. For further consideration, social capital and community identity together posed significant and positive direct influences on life satisfaction. With respect to the mediation effect, residents’ community engagement with perceived flow experience exhibited indirect effects on life satisfaction through social capital; perceived flow experience and green area exhibited indirect effects on life satisfaction through community identity; community engagement posed an indirect chain mediation effect on life satisfaction via social capital and community identity. Therefore, hypotheses H1a, H1b, and H1c were confirmed (see Table 8); H2 (H2a and H2b) and H3 (H3b and H3c) were validated (see Table 8, see Figure 6). In this final validated model, both social capital and community identity served as mediators on the path from the residents’ community engagement, flow experience, and green area to life satisfaction.

## 5. Conclusions and Discussion

What constitutes a happy life in urban residential communities that people are satisfied with? By considering the physical impact of green area within the community, people’s behavioral engagement within the community, their affective and cognitive mental state of flow experience and community identification, as well as social capital, the purpose of this study was to investigate possible factors that are considered vital for predicting life satisfaction toward the urban community, through a proposed model via four groups of hypotheses, and from a comprehensive perspective of physical, psychological, and social aspects. In general, our proposed conceptual model was confirmed, in whole or in part: First, positive and significant correlations were found among green area, community engagement, flow experience, community identification, social capital, and life satisfaction. Second, social capital mediated the path of community engagement to life satisfaction; it also mediated the path of flow experience to life satisfaction. Third, community identity mediated the path of flow to life satisfaction; it also mediated the path of green area to life satisfaction. Finally, social capital posed an indirect effect on life satisfaction through its influence on community identity. Together, these findings enrich the literature on factors influencing urban residential community life satisfaction through a broad perspective that combines different research disciplines within environmental psychology, positive psychology, sociology, and community psychology. Our validated model provides theoretical guidance for policymakers, practitioners, urban community designers, and community managers. The creation of a socially cohesive community in which green areas are vegetated, inviting public spaces where decorated green is arranged, encourages residents to enjoy physical exercise, to interact with neighbors, and to have social activities. Therefore, it helps strengthen urbanites’ feelings of attachment and identification to the residential neighborhood community, so as to increase their life satisfaction toward their residential community.

### 5.1. Positive Direct Influence of Community Engagement, Flow Experience, and Green Area on Life Satisfaction

In terms of urban residential community life satisfaction predictors, and among psychological (affective, cognitive, and behavioral) and physical (e.g., green area) ones, the findings generated from the present dataset confirm our basic model (see H1): community engagement, flow, and green area all played positive and significant contributive roles in explaining people’s life satisfaction toward their residential community.

First and specifically for H1a, our findings are consistent with previous work on the predictor of community engagement to life satisfaction of the community-dwelling older adults’ [51], and extend the prior work to a more general population. Participants actively engaged in community activities, being involved in intrinsically social and dynamic processes, which facilitated communication and interaction with other community members [10,48], increasing “social mobility” towards social empowerment [49]. Thus, engaging in community activities as a community organizational behavior would help build up close connections with other members within this community [48], help improve the level of welfare, promote public trust, eliminate health disparities, and protect environment [50]. The result being that all of these benefits and advantages would facilitate life satisfaction of community residents. In another words, community engagement as a positive organizational behavior is positively associated with life satisfaction toward urban residential community.

Our results for H2b on flow and life satisfaction also confirm prior works and expand research contexts from workplace [45,74] and educational settings [75] by investigating an urban residential community that was rarely studied, both in literature of flow and in community psychology.

Finally, our results confirm H1c on the importance of green area and its benefit for life satisfaction, and are consistent with prior works: people who live and work near natural, or more specifically, green areas have better health and higher levels of satisfaction at home and at work [85]; the larger the green area within the community, the higher the life satisfaction associated with residents [21]. Green area is an important recreational facility of residential areas and includes street trees, squares with green elements, parks, and gardens within the residential communities [18,25,26]. Such an inviting setting provides space for walking, jogging, group dancing, sports, and social recreational activities, and therefore could promote mental and physical health [23,27,28,29] and increase residents’ satisfaction with their community, as well as help them to live in a healthier and more natural way in the city [23].

### 5.2. The Mediating Role of Social Capital

Our results extend prior findings of a small but significant positive association between community-based social capital and individual life satisfaction [90], by showing that social capital is positively associated with the residents’ life satisfaction, supporting prior work [46]. In addition to the direct impact on life satisfaction, social capital was also found to play a mediating role on the path of community engagement to life satisfaction, and on the path between flow and life satisfaction. These mediations partially support prior results showing that social capital served as a bridge to life satisfaction in a special group of elderly migrants in China [71]. Such a finding extends the research context from the virtual community to reality (for mobile social media use) in urban residential communities. The innovative result is that social capital fully mediated the relationship between community engagement and life satisfaction, as well as the relationship between flow and life satisfaction, that is, community engagement and flow no longer had any direct impact on life satisfaction when social capital was put into the model as a mediating variable, which confirms hypothesis H2a and H2b. However, social capital did not show any mediating role in the relationship between green area and life satisfaction (H2c). This result can be interpreted as follows: First of all, social capital provides resources that are from family, friends, or organizations, and may gradually accumulate in social interaction via the engagement and the experience of flow, which, in turn, accumulates in the stock of social capital that is associated with life satisfaction [71]. Second, as such active engagement in community activities and positive enjoyable experience of flow with other community members could enhance self-worth and self-value of residents, it could also reduce their loneliness [133]; these experiences also contribute to the accumulation of social capital, which is linked to life satisfaction. In our study, social capital mediated only the path of the psychological factors (community engagement and flow) to life satisfaction, whereas it did not mediate the path of the physical factor (green area) to life satisfaction.

### 5.3. The Mediating Role of Community Identity

In extending our prior studies on the association between the perceived residential environment quality indicators and neighborhood attachment [13,18], our results confirm that the green area is positively associated with community identity, which is somewhat parallel to recent work on the importance of urban green areas for community members’ sense of belongingness [134,135], and confirm the significant role green open space plays in building residents’ community identity through shared memories and communally engaged activities [58,96]. Findings on the path of flow experience to community identity extend our prior work on the flow–place identity association [35], and to a more specific geographically locative place. Our results confirming H1a reveal that when residents exhibit a higher degree of recognition of their neighborhood residential area, they convey a stronger sense of attachment and identification, they indicate a higher degree of community identity [53,55,56], and they show a higher degree of life satisfaction toward their residential community. From the social identity perspective [61], when a community resident regards himself as one of the community members, he or she may adopt a positive stereotype defined by this community society [62], and this social self-stereotyping process may lead to community identity. The resident’s flow experience plays a pivotal role in the formation of the community identity, because residential experience not only increases the opportunities for interactions among community members, it also provides a temporal background for connecting important life events with residential communities [63], and therefore helps increase life satisfaction, supporting H3b and H3c. The novel finding that psychological flow is positively and significantly associated with life satisfaction via community identity (H3b) expands the research topic dealing with the application of flow theory into urban community. Our innovative finding on the mediation effect of community identity between physiological green areas and life satisfaction (H3c), provides empirical evidence for the possible indirect pathways through which the physical residential environment can affect psychological outcomes like subjective well-being. Such influence was mediated by strengthening the sense of community, and expands the prior work from a specific group of the elderly to a much more general population [12].

### 5.4. The Influence of Social Capital on Community Identity

Empirical findings that social capital exerted a positive influence on community identity (H4) were parallel to some of prior studies on the relationship between social capital and social identity [133], on the relationship between social capital and personal identity [101,134], and on the relationship between social capital and professional identity [97]. We may conclude that the relationship between social capital and identity can also be applied to the community, from the perspective of social identity theory that considers an individual’s aspect of identity is a reflection of his or her sense of who he or she is based on his or her group memberships [135], as well as from the tripartite model of place attachment that deals with the relationship between a person and a place. The urban community residents’ social capital stock determines how many resources they can obtain from their community; this suggests that residents with increasingly abundant social capital stock are inclined to hold a higher identification to their residential neighborhood.

### 5.5. Limits and Future Research

In our study, great efforts have been made to avoid the inadequacies. However, some limits still need to be addressed for future work. First, such a cross-sectional survey dataset can hardly make absolute cause–effect inference. For instance, residents who experience more intense flow may exhibit higher community identity, and perceive stronger life satisfaction. However, this can be turned around to suggest that those who highly identify with their community are more inclined to experience flow when engaging in community-based activities, and as long as there is enjoyment from frequent and intensified flow, they are more satisfied with their life within their community. Therefore, a longitudinal design that tests different relationships among these variables is welcome in future work. Second, regarding the generalization of the present findings, our study was conducted solely in central urban residential communities in Chengdu, Sichuan province, a city located in southwest China, thus, future work will benefit from including more cities or making comparative studies with rural residential communities in China. Still, a cross-cultural study that explores such relations within different capital cities in varying nations is worth trying. Third, as our sample is composed of homeowners and renters, of which over 60% are homeowners, this may explain our findings on stronger community identity [136]. Future work may focus on renters’ community identity based on other predictive variables in our work. Finally, it should be noted that there are many factors affecting life satisfaction of urban community residents beyond flow, green areas, social capital, and community identity. Therefore, more factors, along with their influencing paths on life satisfaction, remain to be explored via the structure equation modeling approach. Future work will also be beneficial for exploring possible potential influencing factors via qualitative comparative analysis

## Figures and Tables

**Table 1:** Social demographic features of participants (*N* = 508).

Category	Options	Responses	Response Rate
Gender	Male	237	46.7%
	Female	271	53.3%
Age	< = 20	106	20.9%
	21–30	233	45.9%
	31–40	94	18.5%
	41–50	31	6.1%
	51–60	20	3.9%
	> = 61	24	4.7%
Educational degree	High school	117	23.0%
	Bachelor	341	67.1%
	Master	48	9.4%
	Ph.D	2	0.4%
Living time	<1	13	2.6%
	1–5	227	44.7%
	6–10	78	15.4%
	11–15	29	5.7%
	16–20	60	11.8%
	>20	101	19.9%
Living areas	1. Pidu District	32	6.3%
	2. Xindu District	31	6.1%
	3. Qingbaijiang District	28	5.5%
	4. Longquanyi District	39	7.7%
	5. Tianfu New District	31	6.1%
	6. Shuangliu District	35	6.9%
	7. Wenjiang District	42	8.3%
	8. Jinniu District	80	15.7%
	9. Chenghua District	49	9.6%
	10. Jinjiang District	31	6.1%
	11. Gaoxin District	42	8.3%
	12. Wuhou District	33	6.5%
	13. Qingyang District	35	6.9%
House-ownership	Owner	311	61.2%
	Tenement	197	38.8%
Total		508	100.0%

**Table 2:** Test of construct validity and reliability.

Items	Factor Loading	Bartlett’s Test	KMO	Cronbach’s alpha	AVE	C.R.	Variance (%)
CE 1	0.893	0.000	0.828	0.904	0.726	0.913	78.121
CE 2	0.924						
CE 3	0.876						
CE 4	0.696						
GA 1	0.801	0.000	0.868	0.889	0.628	0.893	69.738
GA 2	0.866						
GA 3	0.917						
GA 4	0.674						
GA 5	0.674						
CI 1	0.587	0.000	0.827	0.867	0.539	0.852	65.348
CI 2	0.691						
CI 3	0.75						
CI 4	0.803						
CI 5	0.817						
SC 1	0.798	0.000	0.803	0.9	0.624	0.891	71.399
SC 2	0.72						
SC 3	0.69						
SC 4	0.882						
SC 5	0.84						
Flow 1	0.573	0.000	0.749	0.757	0.459	0.767	58.279
Flow 2	0.81						
Flow 3	0.748						
Flow 4	0.539						
LS 1	0.756	0.000	0.845	0.857	0.521	0.844	63.638
LS 2	0.697						
LS 3	0.78						
LS 4	0.653						
LS 5	0.715						

Note: CE—community engagement; GA—green area; CI—community identity; SC—social capital; LS—life satisfaction; KMO—Kaiser-Mayer-Orkin; AVE—Average Variance Extraction; C.R.—Composite Reliability.

**Table 3:** Descriptive statistics on variables.

Variable	Mean	SD	Variance	Skewness		Kurtosis	
	Statistics	Statistics	Statistics	Statistics	SE	Statistics	SE
CE	2.6	1.22044	1.489	0.260	0.108	−0.993	0.216
GA	3.45	0.95143	0.905	−0.162	0.108	−0.393	0.216
CI	3.31	0.85641	0.733	−0.310	0.108	0.243	0.216
SC	2.45	1.01671	1.034	0.266	0.108	−0.450	0.216
Flow	3.25	0.78226	0.612	−0.143	0.108	0.649	0.216
LS	2.96	0.85818	0.736	0.016	0.108	0.047	0.216

Note: CE—community engagement; GA—green area; CI—community identity; SC—social capital; LS—life satisfaction.

**Table 4:** Descriptive statistics and correlations between variables.

Variable	Mean	SD	CE	GA	CI	SC	Flow	LS
CE	2.60	1.22	1					
GA	3.45	0.95	0.078	1				
CI	3.31	0.86	0.255 **	0.564 **	1			
SC	2.45	1.02	0.633 **	0.142 **	0.361 **	1		
Flow	3.25	0.78	0.338 **	0.342 **	0.602 **	0.435 **	1	
LS	2.96	0.86	0.328 **	0.313 **	0.455 **	0.503 **	0.454 **	1

Note: **, *p* < 0.005; CE—community engagement; GA—green area; CI—community identity; SC—social capital; LS—life satisfaction.

**Table 5:** Fitting indices for default model.

Model	X^2^/df	RMSEA	SRMR	NNFI	GFI	IFI	CFI	AIC	ECVI	*p*
Basic model	1.975	0.044	0.0402	0.970	0.950	0.976	0.976	338.891	0.668	0.000
Mediation model	1.855	0.041	0.0436	0.963	0.923	0.969	0.969	766.186	1.511	0.000

**Table 6:** Regression coefficients of the basic model.

Hypothesis	Path	Standardized Estimate	Estimate	S.E.	C.R.	*p*	Decision	Remarks
H1a	Flow → LS	0.376	0.519	0.082	6.309	***	Supported	Confirmative
H1b	CE → LS	0.235	0.199	0.041	4.856	***	Supported	Confirmative
H1c	GA → LS	0.251	0.266	0.052	5.156	***	Supported	Confirmative

Note: ***, *p* < 0.001

**Table 7:** Regression coefficients of the mediation model.

Path	Standardized Estimate	Estimate	S.E.	C.R.	*p*
CE → SC	0.648	0.686	0.054	12.695	***
GA→ SC	0.032	0.041	0.048	0.839	0.401
Flow → SC	0.158	0.292	0.081	3.594	***
Flow → CI	0.461	0.68	0.083	8.155	***
CE → CI	−0.05	−0.042	0.044	−0.967	0.334
GA → CI	0.478	0.488	0.047	10.322	***
SC → CI	0.208	0.166	0.044	3.759	***
SC → LS	0.368	0.254	0.048	5.344	***
CE → LS	−0.027	−0.02	0.043	−0.463	0.643
GA → LS	0.064	0.057	0.054	1.051	0.293
Flow → LS	0.114	0.145	0.091	1.604	0.109
CI → LS	0.373	0.323	0.081	4.003	***

Note: ***, *p* < 0.001; CE—community engagement; GA—green area; CI—community identity; SC—social capital; LS—life satisfaction.

**Table 8:** The bootstrap test of the mediation model.

Hypothesis	Path	Effect Value	Bias-Corrected Percentile		*p*	Decision	Remarks
			Lower	Upper			
H1a	CE → LS	−0.027	−0.09	0.057	0.657	Rejected	Confirmative
H1b	Flow → LS	0.114	−0.075	0.367	0.260	Rejected	Confirmative
H1c	GA → LS	0.064	−0.033	0.157	0.287	Rejected	Confirmative
H2a	CE → SC → LS					Supported	Innovative
	CE → SC	0.648	0.603	0.782	0.000	Supported	
	SC → LS	0.368	0.179	0.341	0.000	Supported	
H2b	Flow → SC → LS					Supported	Innovative
	Flow → SC	0.158	0.159	0.413	0.001	Supported	
	SC → LS	0.368	0.179	0.341	0.000	Supported	
H2c	GA → SC → LS					Rejected	Innovative
	GA → SC	0.032	−0.039	0.127	0.388	Rejected	
	SC → LS	0.368	0.179	0.341	0.000	Supported	
H3a	CE → CI → LS					Rejected	Innovative
	CE → CI	−0.05	−0.107	0.024	0.280	Rejected	
	CI → LS	0.373	0.167	0.492	0.001	Supported	
H3b	Flow → CI → LS					Supported	Innovative
	Flow → CI	0.461	0.53	0.857	0.001	Supported	
	CI → LS	0.373	0.167	0.492	0.001	Supported	
H3c	GA → CI → LS					Supported	Innovative
	GA → CI	0.478	0.392	0.591	0.000	Supported	
	CI → LS	0.373	0.167	0.492	0.001	Supported	
H4	SC → CI	0.208	0.099	0.240	0.000	Supported	Confirmative

Note: CE—community engagement; GA—green area; CI—community identity; SC—social capital; LS—life satisfaction.

## References

[1] Diner E., Oishi S., Lucas R.E. (2003). Personality, culture, and subjective well-being: Emotional and cognitive evaluations of life. Ann. Rev. Psychol..

[2] Diener E., Suh E.M., Lucas R.E., Smith H.L. (1999). Subjective well-being: Three decades of progress. Psychol. Bull..

[3] Leto I.V., Petrenko E.N., Slobodskaya H.R. (2019). Life satisfaction in Russian primary schoolchildren: Links with personality and family environment. J. Happiness Stud..

[4] Udayar S., Urbanaviciute I., Massoudi K., Rossier J. (2020). The role of personality profiles in the longitudinal relationship between work-related well-being and life satisfaction among working adults in Switzerland. Eur. J. Personal..

[5] Proctor C.L., Alex Linley P., Maltby J. (2008). Youth life satisfaction: A review of the literature. J. Happiness Stud..

[6] Salmani S., Biderafsh A., Aliakbarzadeh Z.A. (2019). The relationship between spiritual development and life satisfaction among students of Qom University of medical sciences. J. Relig. Health.

[7] Freire T., Ferreira G. (2020). Do I need to be positive to be happy? Considering the role of self-esteem, life satisfaction, and psychological distress in Portuguese adolescents’ subjective happiness. Psychol. Rep..

[8] Lissitsa S., Chachashvili-Bolotin S. (2016). Life satisfaction in the internet age—Changes in the past decade. Comput. Hum. Behav..

[9] Abass Z.I., Tucker R. (2018). Residential satisfaction in low-density Australian suburbs: The impact of social and physical context on neighbourhood contentment. J. Environ. Psychol..

[10] Li L., Loo B.P. (2017). Mobility impairment, social engagement, and life satisfaction among the older population in China: A structural equation modeling analysis. Qual. Life Res..

[11] Mhaoláin A.M.N., Gallagher D., Connell H.O., Chin A.V., Bruce I., Hamilton F., Teehee E., Coen R., Coakley D., Cunningham C. (2012). Subjective well-being amongst community-dwelling elders: What determines satisfaction with life? Findings from the Dublin healthy aging study. Int. Psychogeriatr..

[12] Zhang Z., Zhang J. (2017). Perceived residential environment of neighborhood and subjective well-being among the elderly in China: A mediating role of sense of community. J. Environ. Psychol..

[13] Bonaiuto M., Aiello A., Perugini M., Bonnes M., Ercolani A.P. (1999). Multidimensional perception of residential environment quality and neighbourhood attachment in the urban environment. J. Environ. Psychol..

[14] Bonaiuto M., Fornara F., Alves S., Ferreira I., Mao Y., Moffat E., Rahimi L. (2015). Urban environment and well-being: Cross-cultural studies on perceived residential environment quality indicators (PREQIs). Cogn. Proc..

[15] Bonnes M., Bonaiuto M., Ercolani A.P. (1991). Crowding and residential satisfaction in the urban environment: A con- textual approach. Environ. Behav..

[16] Bonnes M., Scopelliti M., Fornara F., Carrus G., Steg L., van den Berg A.E., de Groot J.I.M. (2012). Urban environmental quality. Environmental Psychology: An Introduction.

[17] Fornara F., Bonaiuto M., Bonnes M. (2010). Cross-validation of abbreviated perceived residential environment quality (PREQ) and neighbourhood attachment (NA) indicators. Environ. Behav..

[18] Mao Y., Fornara F., Manca S., Bonnes M., Bonaiuto M. (2015). Perceived residential environment quality indicators and neighborhood attachment: A confirmation study on a Chinese sample in Chongqing. Psych. J..

[19] Bonaiuto M., Fornara F., Stein J. (2017). Residential satisfaction and perceived urban quality. Reference Module in Neuroscience and Biobehavioral Psychology.

[20] White M.P., Alcock I., Wheeler B.W., Depledge M.H. (2013). Would you be happier living in a greener urban area? A fixed-effects analysis of panel data. Psychol. Sci..

[21] White M.P., Alcock I., Grellier J., Wheeler B.W., Hartig T., Warber S.L., Fleming L.E. (2019). Spending at least 120 minutes a week in nature is associated with good health and wellbeing. Sci. Rep..

[22] Hartig T., Spielberger C. (2004). Restorative Environments. Encyclopedia of Applied Psychology.

[23] Hartig T., Kahn P.H. (2016). Living in cities, naturally. Science.

[24] Krellenberg K., Welz J., Reyes-Packe S. (2014). Urban green areas and their potential for social interaction—A case study of a socio-economically mixed neighbourhood in Santiago de Chile. Habitat Int..

[25] Masoudinejad S., Hartig T. (2020). Window view to the sky as a restorative resource for residents in densely populated cities. Environ. Behav..

[26] Carrus G., Scopelliti M., Lafortezza R., Colangelo G., Ferrini F., Salbitano F., Sanesi G. (2015). Go greener, feel better? The positive effects of biodiversity on the well-being of individuals visiting, urban and peri-urban green areas. Landsc. Urban Plan..

[27] Campos-Sanchez F.S., Valenzuela-Montes L.M., Abarca-Alvarez F.J. (2019). Evidence of green areas, cycle infrastructure and attractive destinations working together in development on urban cycling. Sustainability.

[28] Dadvand P., Bartoll X., Basagana X., Dalmau-Bueno A., Martinez D., Ambros A., Nieuwenhuijsen M.J. (2016). Green spaces and general health: Roles of mental health status, social support, and physical activity. Env. Int..

[29] Persson A., Moller J., Engstrom K., Sundstrom M.L., Nooijen C.F.J. (2019). Is moving to a greener or less green area followed by changes in physical activity?. Health Place.

[30] Valente D., Pasimeni M.R., Petrosillo I. (2020). The role of green infrastructures in Italian cities by linking natural and social capital. Ecol. Indic..

[31] Žlender V., Gemin S. (2020). Testing urban dwellers’ sense of place towards leisure and recreational peri-urban green open spaces in two European cities. Cities.

[32] Cheng J.C.-H., Monroe M.C. (2010). Connection to nature: Children’s affective attitude toward nature. Environ. Behav..

[33] Fernandez-Portero C., Alarcon D., Padura A.B. (2017). Dwelling conditions and life satisfaction of older people through residential satisfaction. J. Environ. Psychol..

[34] Roster C.A., Ferrari J.R., Peter Jurkat M. (2016). The dark side of home: Assessing possession ‘clutter’ on subjective well-being. J. Environ. Psychol..

[35] Bonaiuto M., Mao Y., Roberts S., Psalti A., Ariccio S., Ganucci Cancellieri U., Csikszentmihalyi M. (2016). Optimal experience and personal growth: Flow and the consolidation of place identity. Front. Psychol..

[36] Csikszentmihalyi M. (1975). Beyond Boredom and Anxiety.

[37] Csikszentmihalyi M. (1990). Flow: The Psychology of Optimal Experience.

[38] Csikszentmihalyi M., Csikszentmihalyi I. (1988). Optimal Experience.

[39] Mao Y., Yang R., Bonaiuto M., Ma J., Harmat L. (2020). Can flow alleviate anxiety? The roles of academic self-efficacy and self-esteem in building psychological sustainability and resilience. Sustainability.

[40] Abuhamdeh S. (2020). Investigating the “flow” experience: Key conceptual and operational issues. Front. Psychol..

[41] Hopp T., Barker V. (2016). Investigating the influence of age, social capital affinity, and flow on positive outcomes reported by e-commerce site users. Behav. Inf. Technol..

[42] Liu T.S., Csikszentmihalyi M. (2020). Flow among introverts and extraverts in solitary and social activities. Personal. Individ. Differ..

[43] Csikszentmihalyi M. (1997). Finding Flow: The Psychology of Engagement with Everyday Life.

[44] Csikszentmihalyi M., LeFevre J. (1989). Optimal experience in work and leisure. J. Personal. Soc. Psychol..

[45] Ramsey J.R., Lorenz M.P. (2019). Every flow has its ebb: The impact of flow on work–family conflict and adjustment in global careers. Hum. Res. Manag. J..

[46] Pang B.N. (2020). Engaging Bourdieu’s habitus with Chinese understandings of embodiment: Knowledge flows in health and physical education in higher education in Hong Kong. Educ. Philos. Theory.

[47] Chen L.H., Ye Y.C., Chen M.Y., Tung I.W. (2010). Alegria! Flow in leisure and life satisfaction: The mediating role of event satisfaction using data from an acrobatic show. Soc. Indic. Res..

[48] Johnston K.A., Lane A.B. (2018). Building relational capital: The contribution of episodic and relational community engagement. Public Relat. Rev..

[49] Hoe K.C., Abd Wahab H., Abu Bakar S.H., Islam M.R. (2017). Community participation for rural poverty alleviation: A case of the Iban community in Malaysia. Int. Soc. Work.

[50] Hayes J.E., Fisher R.M., Stevenson R.J., Stuetz R.M. (2019). Investigation of non-community stakeholders regarding community engagement and environmental malodour. Sci. Total Environ..

[51] Bertelli-Costa T., Neri A.L. (2019). Life satisfaction and participation among community-dwelling older adults: Data from the FIBRA study. J. Health Psychol..

[52] Stets J.E., Biga C.F. (2003). Bringing identity theory into environmental sociology. Sociol. Theory.

[53] Grey C., O’Toole M. (2020). The placing of identity and the identification of place: “Place-identity” in community lifeboating. J. Manag. Inq..

[54] Twigger-Ross C., Bonaiuto M., Breakwell G., Bonnes M., Lee T., Bonaiuto M. (2003). Identity theories and environmental psychology. Psychological Theories for Environmental Issue.

[55] Proshansky H., Fabian A., Kaminoff R. (1983). Place-identity: Physical world socialization of the self. J. Environ. Psychol..

[56] Wynveen C., Schneider I., Arnberger A., Cottrell S.P., von Ruschkowski E. (2020). Integrating place attachment into management frameworks: Exploring place attachment across the recreation opportunity spectrum. Environ. Manag..

[57] Scannell L., Gifford R. (2010). The relations between natural and civic place attachment and pro-environmental behavior. J. Environ. Psychol..

[58] Putra B.D., Horne R., Hurley J. (2019). Place, space and identity through greening in Kampung Kota. J. Reg. City Plan..

[59] Reese G., Oettler L.M.S., Katz L.C. (2019). Imagining the loss of social and physical place characteristics reduces place attachment. J. Environ. Psychol..

[60] Christiaanse S., Haartsen T. (2020). Experiencing place-change: A shared sense of loss after closure of village facilities. J. Environ. Psychol..

[61] Tajfel H., Turner J., Austin W.G., Worchel S. (1979). An integrative theory of intergroup conflict. The Social Psychology of Intergroup Relations.

[62] Su L., Huang S., Nejati M. (2019). Perceived justice, community support, community identity and residents’ quality of life: Testing an integrative model. J. Hosp. Tour. Manag..

[63] Paskett E.D., Young G.S., Bernardo B.M., Washington C., DeGraffinreid C., Fisher J.L., Huerta T.R. (2019). Correlates of rural, appalachian, and community identity in the cities cohort. J. Rural Health.

[64] Bourdieu P., Richardson J.G. (1986). Forms of capital. Handbook of Theory and Research for the Sociology of Education.

[65] Coleman J.S. (1988). Social capital in the creation of human capital. Am. J. Sociol..

[66] Baker W.E. (1990). Market networks and corporate behavior. Am. J. Sociol..

[67] Putnam R.D. (1993). The prosperous community: Social capital and public life. Am. Prospect..

[68] Portela M., Neira I., Salinas-Jiménez M.D.M. (2013). Social capital and subjective wellbeing in Europe: A new approach on social capital. Soc. Indic. Res..

[69] Van Oorschot W., Arts W. (2005). The social capital of European welfare states: The crowding out hypothesis revisited. J. Eur. Soc. Policy.

[70] Ruef M., Kwon S.W. (2016). Neighborhood associations and social capital. Soc. Forces.

[71] Zhao L., Liang C.Y., Gu D.X. (2020). Mobile social media use and trailing parents’ life satisfaction: Social capital and social integration perspective. Int. J. Aging Hum. Dev..

[72] Ji X.W., Chui C.H.K., Ni S.G., Dong R. (2020). Life satisfaction of rural migrant workers in urban China: The roles of community service participation and identity integration. J. Soc. Serv. Res..

[73] Takahashi K., Nguyen T.M.T., Poudel K.C., Sakisaka K., Jimba M., Yasuoka J. (2011). Social capital and life satisfaction: A cross-sectional study on persons with musculoskeletal impairments in Hanoi, Vietnam. BMC Public Health.

[74] Datu J.A.D., Mateo N.J. (2017). Work-related flow dimensions differentially predict anxiety, life satisfaction, and work longevity among Filipino counselors. Curr. Psychol..

[75] Olcar D., Rijavec M., Golub T.L. (2019). Primary school teachers’ fife satisfaction: The role of life goals, basic psychological needs and flow at work. Curr. Psychol..

[76] Habe K., Biasutti M., Kajtna T. (2019). Flow and satisfaction with life in elite musicians and top athletes. Front. Psychol..

[77] Hirao K., Kobayashi R., Okishima K., Tomokuni Y. (2012). Flow experience and health-related quality of life in community dwelling elderly Japanese. Nurs. Health Sci..

[78] Kang J.-W., Lee H., Namkung Y. (2018). The impact of restaurant patrons’ flow experience on SNS satisfaction and offline purchase intentions. Int. J. Contemp. Hosp. Manag..

[79] Vitters J. (2003). Flow versus life satisfaction: A projective use of cartoons to illustrate the difference between the evaluation approach and the intrinsic motivation approach to subjective quality of life. J. Happiness Stud..

[80] Cetin C., Karafaki F.C. (2020). The influence of green areas on city-dwellers’ perceptions of air pollution: The case of Nigde city center. J. Environ. Biol..

[81] Gidlof-Gunnarsson A., Ohrstrom E. (2007). Noise and well-being in urban residential environments: The potential role of perceived availability to nearby green areas. Landsc. Urban Plan..

[82] Dzhambov A.M., Hartig T., Tilov B., Atanasova V., Makakova D.R., Dimitrova D.D. (2019). Residential greenspace is associated with mental health via intertwined capacity-building and capacity-restoring pathways. Environ. Res..

[83] Vujcic M., Tomicevic-Dubljevic J., Zivojinovic I., Toskovic O. (2019). Connection between urban green areas and visitors’ physical and mental well-being. Urban For. Urban Green..

[84] Constantinescu M., Orindaru A., Caescu S.C., Pachitanu A. (2019). Sustainable development of urban green areas for quality of life improvement-argument for increased citizen participation. Sustainability.

[85] McFarland A.L. (2017). The relationship between the use of green spaces and public gardens in the work place on mental well-being, quality of life, and job satisfaction for employees and volunteers. Horttechnology.

[86] Collins C.R., Neal J.W., Neal Z.P. (2014). Transforming individual civic engagement into community collective efficacy: The role of bonding social capital. Am. J. Commun. Psychol..

[87] Kaur P., Dhir A., Chen S., Rajala R. (2016). Flow in context: Development and validation of the flow experience instrument for social networking. Comput. Hum. Behav..

[88] Hong A., Sallis J.F., King A.C., Conway T.L., Saelens B., Cain K.L., Frank L.D. (2018). Linking green space to neighborhood social capital in older adults: The role of perceived safety. Soc. Sci. Med..

[89] Kingsley J., Foenander E., Bailey A. (2020). “It’s about community”: Exploring social capital in community gardens across Melbourne, Australia. Urban For. Urban Green..

[90] Hoogerbrugge M.M., Burger M.J. (2018). Neighborhood-Based social capital and life satisfaction: The case of Rotterdam, The Netherlands. Urban Geogr..

[91] Chuang Y.W. (2020). Promoting consumer engagement in online communities through virtual experience and social identity. Sustainability.

[92] Erentaite R., Vosylis R., Gabrialaviciute I., Raiziene S. (2018). How does school experience relate to adolescent identity formation over time? Cross-lagged associations between school engagement, school burnout and identity processing styles. J. Youth Adolesc..

[93] Lannegrand-Willems L., Chevrier B., Perchec C., Carrizales A. (2018). How is civic engagement related to personal identity and social identity in late adolescents and emerging adults? A person-oriented approach. J. Youth Adolesc..

[94] Mao Y., Roberts S., Pagliaro S., Csikszentmihalyi M., Bonaiuto M. (2016). Optimal experience and optimal identity: A multinational study of the associations between flow and social identity. Front. Psychol..

[95] Orta A., Sicilia A., Fernandez-Balboa J.M. (2017). Relationship between flow and athletic identity: The case of three elite sportsmen. Quest.

[96] Kim J., Kaplan R. (2004). Physical and psychological factors in sense of community: New urbanist Kentlands and nearby orchard village. Environ. Behav..

[97] Jensen D.H., Jetten J. (2015). Bridging and bonding interactions in higher education: Social capital and students’ academic and professional identity formation. Front. Psychol..

[98] Heidari E., Salimi G., Mehrvarz M. (2020). The influence of online social networks and online social capital on constructing a new graduate students’ professional identity. Interact. Learn. Environ..

[99] Ho T.K., Lin Y.T. (2016). The effects of virtual communities on group identity in classroom management. J. Educ. Comput. Res..

[100] Kim S.K. (2015). The effects of family and school social capital on youths’ career identity. J. Soc. Child Welf..

[101] Kerpelman J., White L. (2016). Interpersonal identity and social capital: The importance of commitment for low income, rural, African American adolescents. J. Black Psychol..

[102] Flannery R. Shanghai Tops New Forbes China Ranking of Best Cities for Living. http://www.ce.cn/cysc/fdc/fc/201902/28/t20190228_31586219.

[103] Kuo L. Inside Chengdu: Can China’s Megacity Version of the Garden City Work? The Guardian. ISSN 02613077. https://www.lboro.ac.uk/gawc/world2020t.html.

[104] Diener E., Emmons R.A., Larsen R.J., Griffin S. (1985). The satisfaction with life scale. J. Personal. Assess..

[105] Pavot W., Diener E. (1993). Review of the satisfaction with life scale. Psychol. Assess..

[106] Lyubomirsky S., Lepper H. (1999). A measure of subjective happiness: Preliminary reliability and construct validation. Soc. Indic. Res..

[107] Wakefield J.R.H., Sani F., Madhok V., Norbury M., Dugard P., Gabbanelli C., Poggesi F. (2017). The Relationship Between Group Identification and Satisfaction with Life in a Cross-Cultural Community Sample. J. Happiness Stud..

[108] Cronbach L.J. (1951). Coefficient alpha and the internal structure of tests. Psychometrika.

[109] Mao Y., Roberts S., Bonaiuto M., Harmat L., Ørsted Andersen F., Ullén F., Wright J., Sadlo G. (2016). Optimal experience and optimal identity: A multinational examination at the personal identity level. Flow Experience: Empirical Research and Applications.

[110] Mao Y., Lai Y., Luo Y., Liu S., Du Y., Zhou J., Bonaiuto M. (2020). Apple or Huawei: Understanding flow, brand image, brand identity, brand personality and purchase intention of smartphone. Sustainability.

[111] Waterman A.S., Schwartz S.J., Goldbacher E., Green H., Miller C., Philip S. (2003). Predicting the subjective experience of intrinsic motivation: The roles of self-determination, the balance of challenges and skills, and self-realization values. Personal. Soc. Psychol. Bull..

[112] Waterman A.S. (2005). When effort is enjoyed: Two studies of intrinsic motivation for personally salient activities. Motiv. Emot..

[113] Waterman A.S., Schwartz S.J., Conti R. (2008). The implications of two conceptions of happiness (hedonic enjoyment and eudaimonia) for the understanding of intrinsic motivation. J. Happiness Stud..

[114] Herrero J., Gracia E. (2007). Measuring perceived community support: Factorial structure, longitudinal invariance, and predictive validity of the PCSQ (Perceived Community Support Questionnaire). J. Commun. Psychol..

[115] Jiménez T.I., Musitu G., Ramos M.J., Murgui S. (2010). Community involvement and victimization at school: An analysis through family, personal and social adjustment. J. Commun. Psychol..

[116] Bergefurt L., Kemperman A., van den Berg P., Borgers A., van der Waerden P., Oosterhuis G., Hommel M. (2019). Loneliness and life satisfaction explained by public-space use and mobility patterns. Int. J. Environ. Res. Public Health.

[117] De Silva M.J., Huttly S.R., Harpham T., Kenward M.G. (2007). Social capital and mental health: A comparative analysis of four low income countries. Soc. Sci. Med..

[118] Trudy H., Emma G., Elizabeth T. (2002). Measuring social capital within health surveys: Key issues. Health Policy Plan..

[119] Gausman J., Langer A., Austin S.B., Subramanian S.V. (2019). Contextual Variation in Early Adolescent Childbearing: A Multilevel Study From 33,822 Communities in 44 Low- and Middle-Income Countries. J. Adolesc. Health.

[120] Proshansky H.M. (1978). The self and the city. Environ. Behav..

[121] Bonaiuto M., Bonnes M., Wapner S., Demick J., Yamamoto T., Minami H. (2000). Social-psychological approaches in environment-behavior studies. Identity theories and the discursive approach. Theoretical Perspectives in Environment-Behavior Research: Underlying Assumptions, Research Problems, and Methodologies.

[122] Jorgensen B.S., Stedman R.C. (2001). Sense of place as an attitude: Lakeshore owners’ attitudes toward their properties. J. Environ. Psychol..

[123] Williams D.R., Vaske J.J. (2003). The measurement of place attachment: Validity and generalizability of a psychometric approach. For. Sci..

[124] Kaiser H.F., Rice J. (1974). Little Jiffy, Mark Iv. Educ. Psychol. Meas..

[125] Hu L.-t., Bentler P.M. (1999). Cutoff criteria for fit indexes in covariance structure analysis: Conventional criteria versus new alternatives. Struct. Equ. Model..

[126] Browne M.W., Cudeck R., Bollen K.A., Long J.S. (1993). Alternative ways of assessing model fit. Testing Structural Equation Models.

[127] Kline R.B. (2005). Principles and Practice of Structural Equation Modeling.

[128] Bentler P. (1990). Comparative fit indexes in structural models. Psychol. Bull..

[129] Hsu I.Y., Su T.S., Kao C.S., Shu Y.L., Lin P.R., Tseng J.M. (2012). Analysis of business safety performance by structural equation models. Saf. Sci..

[130] Yang L., Feng Z., Zhao X., Jiang K., Huang Z. (2020). Analysis of the factors affecting drivers’ queue-jumping behaviors in China. Transp. Res. Part F Traffic Psychol. Behav..

[131] Fornell C., Larcker D.F. (1981). Evaluating structural equation models with unobservable variables and measurement error. J. Mark. Res..

[132] Bagozzi R.P., Yi Y. (1988). On the evaluation of structural equation models. Acad. Market. Sci..

[133] Kaye L.K., Kowert R., Quinn S. (2017). The role of social identity and online social capital on psychosocial outcomes in MMO players. Comput. Hum. Behav..

[134] Rugel E.J., Carpiano R.M., Henderson S.B., Brauer M. (2019). Exposure to natural space, sense of community belonging, and adverse mental health outcomes across an urban region. Environ. Res..

[135] Ashforth B.E., Mael F.A. (1989). Social identity theory and the organization. Acad. Manag. Rev..

[136] Hernández B., Hidalgo M.C., Salazar-Laplace M.E., Hess S. (2007). Place attachment and place identity in natives and non-natives. J. Environ. Psychol..

